# Evaluation of Partner Notification Strategies to Improve Syphilis Management in Pregnancy in Blantyre, Malawi: A Mixed-Methods Study

**DOI:** 10.1097/OLQ.0000000000002353

**Published:** 2026-05-27

**Authors:** Kondwani Kaitume Kaunda, Deirdre J. Foley, Michael Marks, Annielisa Majamanda, Monica Patricia Malata, Catherine Bamuya, Chifundo Kondoni, Gladys Membe Gadama, David Lissauer, Chelsea Morroni, Peter MacPherson, Effie Chipeta, Linda Mipando, Brynne Gilmore, Bridget Freyne

**Affiliations:** From the ^*^Department of Public Health, KuHes, Blantyre, Malawi; † School of Medicine, University College Dublin; ‡ Departments of Infection & Global Health, Children’s Health Ireland, Dublin, Ireland; § Clinical Research Department, Faculty of Infectious and Tropical Diseases, London School of Hygiene & Tropical Medicine; ¶ Hospital for Tropical Diseases, University College London Hospital, London, United Kingdom; ∥ Department of Quality, Blantyre District Health Office; ** Center for Reproductive Health, College of Medicine, University of Malawi; †† Malawi Epidemiology and Infection Research Unit (MEIRU); ‡‡ Malawi Liverpool Wellcome Research Program, Blantyre, Malawi; §§ Department of Obstetrics, Queen Elizabeth Central Hospital; ¶¶ Institute of Life Course and Medical Sciences, University of Liverpool, Liverpool Women’s Hospital, Liverpool, ^∥∥^Centre for Reproductive Health, University of Edinburgh, Edinburgh, Scotland, United Kingdom; ^***^Botswana Harvard Health Partnership, Gaborone, Botswana; ††† School of Health & Wellbeing, University of Glasgow, United Kingdom; ‡‡‡ School of Nursing, Midwifery and Health Systems, University College Dublin, Dublin, Ireland.

## Abstract

**Background:**

Congenital syphilis remains a major global health challenge, driven partly by maternal reinfection during pregnancy. Partner notification (PN) is critical to prevent reinfection and reduce mother-to-child transmission, yet evidence on its uptake and effectiveness is limited. We aimed to assess PN coverage, outcomes, and barriers among pregnant women with syphilis in Blantyre, Malawi.

**Methods:**

We conducted a mixed-methods study at 4 primary health centers (Mpemba, Zingwangwa, South Lunzu, and Bangwe). A cross-sectional survey at delivery enrolled women with documented syphilis in the current pregnancy (positive treponemal rapid diagnostic test). PN was defined as informing partners of potential exposure and the need for testing; appropriate partner management was defined as partner testing and treatment according to results. Semistructured interviews with women and healthcare workers were analyzed using reflexive thematic analysis.

**Results:**

Among 131 women, 117/131 (89.3%) reported notifying partners; however, only 76/131 (58.0%) partners were tested, and a similar but nonidentical 76/131 (58.0%) received treatment. Appropriate partner management was completed in 40/131 (30.5%). Partner treatment was associated with being married or cohabiting (74/76, 97.4% vs 43/55, 78.2%; P = 0.0005), but not with age, HIV status, education, or adverse birth outcomes. Qualitative findings identified predominantly patient-led PN, with barriers, including fear of partner reaction, stigma, misinformation, and partner reluctance to access care; facilitators included community sensitization and healthcare worker involvement.

**Conclusions:**

Substantial attrition occurs between notification and partner management, highlighting critical gaps in the cascade. These findings support the need for standardized PN metrics and context-specific, multicomponent interventions addressing health system barriers.

## Abstract 2

We report attrition at multiple points in the PN process. Identified barriers to PN included fear of disclosure, male partner disengagement from antenatal services, and financial and access barriers.

The incidence of syphilis in women of reproductive age and congenital syphilis (CS) has increased globally over the last decade. In the United States, syphilis in birthing parents rose from 87 to 280 per 100,000 births, while Australia has reported a greater than 250% rise in cases amongst women of reproductive age, with disproportionately higher increases seen in indigenous populations.^1,2^ In Malawi, modeling of HMIS data suggests an increasing prevalence of gestational syphilis between 2014 and 2022.^3^ CS continues to exceed elimination targets, with the WHO estimating a global incidence of 523 cases per 100,000 live births in 2022.^3,4^ The highest burden continues to be attributed to the WHO African Region, with an estimated 390,000 adverse birth outcomes, including 150,000 early fetal deaths and stillbirths, and 70,000 neonatal deaths globally each year.^4–6^

Partner treatment is known to directly impact the incidence of CS. In a cohort of 1,541 pregnant women with syphilis in Brazil, successful partner treatment significantly reduced the risk of CS (absolute risk reduction 0.60, 95% confidence interval, 0.55–0.66) and lowered the rates of fetal loss from 21.7% to 4.3%, prematurity from 22% to 10.7%, and low birth weight from 24.4% to 10.7%.^7^ These studies also highlighted the potential for residual risk due to incident maternal infection in late pregnancy, which can still result in adverse outcomes despite partner management (PM). It is evident that it is necessary for clinicians to consider the partner factors throughout the antenatal course for women with gestational syphilis, including active evaluation of these factors at birth.^8^

Partner notification (PN) is a critical element of World Health Organisation’s Elimination of Mother to Child Transmission (WHO’s EMTCT) framework, which describes a holistic approach to maternal, partner, and infant management for prevention of syphilis in pregnancy.^9^ Despite its importance, there is no standardized definition or agreed indicators for PN strategies. Studies on PN among pregnant women with syphilis include 2 randomized controlled trials (RCTs) and 7 cohort studies with a total of 3325 participants, evaluating interventions, including patient-led notification and provider-assisted referral.^10–18^ Strategies associated with higher rates of successful PN involve couple-based interventions, targeted healthcare worker (HCW) education, and SMS reminders. Barriers to PN in this context, which were consistently reported included fear of blame in relationships and limited access to testing and treatment facilities.^10–18^

To our knowledge, this is the first study aimed specifically at PN for syphilis in pregnancy in Malawi. Five prior studies on PN of HIV in pregnancy in Malawi, including 2 RCTs and 3 qualitative studies, offer some important perspectives.^19–23^ The RCTs demonstrated that invitation cards for partners to attend antenatal care (ANC) and couple-based behavioral interventions improved partner attendance and HIV viral suppression rates.^19,20^ Qualitative studies identified barriers such as relationship conflict and PN process failures. Facilitators included trust in healthcare providers and the potential of community-level strategies, including both male peer and couples-based interventions.^21,22^ Strategies for PN for syphilis in pregnancy can be learned from studies in HIV and other STI but must be tailored to the timeframe of pregnancy and the potential to prevent adverse birth outcomes. Malawian national guidance advocates for participant-led notification and testing of partners of individuals with syphilis.^24^

This mixed-methods study comprehensively evaluated PN coverage, including the assessment of both partner testing and partner treatment outcomes, and identified barriers and facilitators that can be overcome or utilized in the design of future interventions to improve PN for syphilis in pregnancy in this context.

## MATERIALS AND METHODS

This was a mixed-methods study, conducted at 4 urban and periurban primary health centers (Mpemba, Zingwangwa, South Lunzu, and Bangwe) in Blantyre district. All are operating within the National Ministry of Health structures and surveillance systems and are staffed by nurses and clinical officers employed by the government. All women who delivered during the 9-month data collection period, from 20 January to 30 September 2021, were screened using delivery registers, and those who were seropositive for syphilis were eligible for inclusion in the study. Health passports were screened to ascertain serological evidence of syphilis in the current pregnancy, as defined by a positive treponemal rapid diagnostic test on finger-prick sampling at ANC. Additionally, quantitative data, including self-reported demographics, marital status, and PN history, were collected using structured questionnaires. Information on HIV status was collected using maternal health passport documentation of an HIV antibody test in the current pregnancy.

PN was defined as the process of informing a partner(s) of their potential syphilis exposure and the need for testing and treatment. Appropriate PM was defined as a partner who was tested and received care appropriate to their test result. Partner treatment (irrespective of partner test result) was also described. Both the coverage and method of PN were recorded. Partners were considered “treated” for syphilis if they received at least 1 injection of benzathine penicillin. If partner treatment status was unknown or unclear, they were considered inadequately treated.

Quantitative data were analyzed using STATA v14.0 (StataCorp LLC, College Station, TX). Descriptive statistics were used to summarize demographics and coverage outcomes. Demographic characteristics of women whose partners were treated compared with those untreated were compared using χ^2^ test of independence for categorical variables and the Mann–Whitney *U* test for continuous variables.

Semistructured qualitative interviews were conducted in Chichewa or English with pregnant women who were seropositive for syphilis and HCWs at ANC, postnatal wards, and STI clinics. All participants were conveniently sampled. Topic guides, specific to participant group, which covered PN knowledge and experiences were used. Interviews were conducted in private settings by trained research assistants, audio-recorded with consent and then transcribed verbatim and translated as necessary. Senior investigators reviewed audio recordings and conducted regular debriefings with interviewers to ensure fidelity to the topic guide and minimize interviewer bias. Data were analyzed in NVivo (version 1.7.2, Lumivero, Denver, CO) using reflexive thematic analysis.^25^ Transcripts were iteratively reviewed to ensure immersion, and coding was performed. Codes were grouped and refined through discussion to achieve consensus, with continuous team-based review, reflexivity, and regular debriefing during both data collection and analysis to enhance consistency and reduce bias in interpretation.

All potential participants were given study information detailing the study, its voluntary nature, and their right to withdraw or not answer at any time before taking informed consent. For those aged <18 years, the participant was given the opportunity to assent, and their legal guardian then completed the formal consent on their behalf. Ethical approval was received from The University of Malawi, College of Medicine Research and Ethics Committee and the University of Liverpool Research Ethics Committee. This study was undertaken with the support of the DIPLOMATIC Global Health Group.^1^

## RESULTS

Over the 9-month period of the study, there were 2730 deliveries across the 4 health centers. Of these women, 1993/2730 (73%) had been tested for syphilis in pregnancy, and 144/1993 (7.2%) had a positive syphilis treponemal rapid diagnostic test result. Of 144 eligible women, 131 agreed to complete the quantitative survey. The median age was 25 years (range 15–50 years), most were married or cohabiting (117/131, 89.3%) and 41/131 (31.3%) reported they were living with HIV, of whom 37/41 (90.2%^2^) were taking ART. Most women (110/131, 83.9%) received syphilis treatment during pregnancy, with treatment initiated at ANC1 in 58/110 (52.7%), ANC2 in 10/110 (9.1%), or ANC3 or beyond in 42/110 (38.2%).

**Figure 1.**
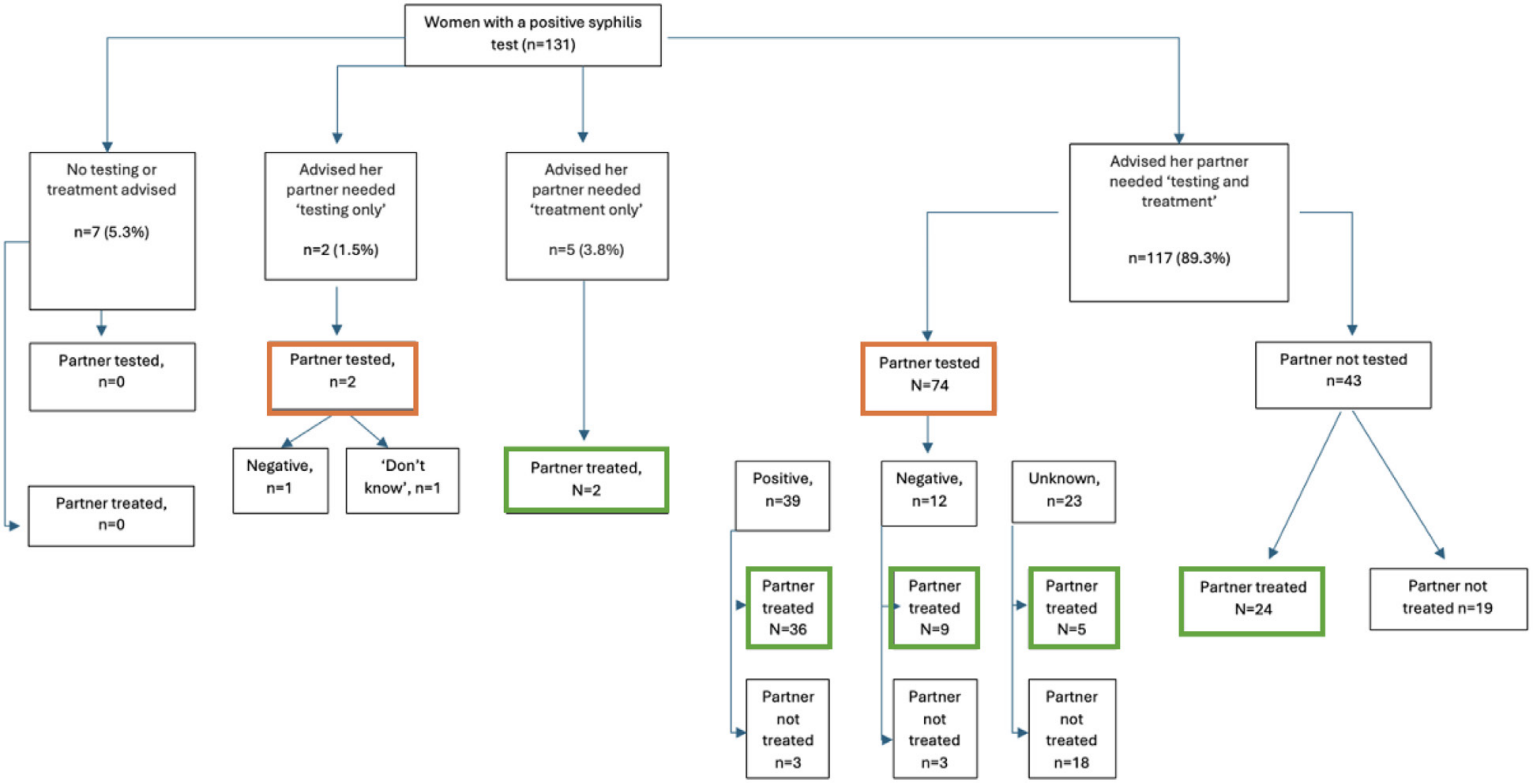
Describes the partner notification and treatment process from point of informing the participant of the need for partner testing and/or treatment. The outcome of partner testing (orange) or partner treatment (green) is outlined.

There was variability in the process and implementation of PN in this cohort (Fig. 1). PN, defined as the process of HCWs informing women of the need for their partners to attend for testing and treatment was reported to be 89.3% (117/131). A further 5 women were advised of the need for partner treatment alone and 2 for testing alone. A total of 76/131 (58%) male partners underwent syphilis testing, and the same proportion (76/131, 58%) received treatment, of note, these were overlapping but nonidentical groups (Fig. 1). It is notable that there were 26 partners treated who were untested and 9 who tested negative but went on to receive treatment. Overall, 40/131 (30.5%) of partners met the definition for appropriate PM. Of 76 partners who received treatment, 52/76 (68.4%) received free treatment at ANC or an STI clinic, 21/76 (27.6%) paid for treatment at an STI clinic or private clinic and for 3/76 (3.9%), payment status was unknown.

Partner treatment was not associated with maternal age (25.5 vs 25 yr, *P* = 0.156), HIV status (23/76, 30.3% vs 18/55, 32.7%; *P* = 0.955) or completion of postprimary education (30/76, 39.5% vs 17/55, 30.9%; *P* = 0.549). Only relationship status, that is, being married or cohabiting (74/76, 97.4% vs 43/55, 78.2%; *P* = 0.0005) was significantly associated with successful partner treatment (Table 1). Overall, 13/131 (9.9%) infants had at least 1 adverse birth outcome (prematurity n = 6, low birth weight n = 5, and stillbirth n = 2), though this was not associated with partner treatment (9/76, 11.8% vs 4/55,7.3%, *P* = 0.3898). Among mothers with an adverse birth outcome, 10/13 (76.9%) women had received 3 doses of IM BPG in pregnancy, of whom 9/10 (90%) had partners who were treated. The remaining 3 women in this group and their partners were untreated.

**TABLE 1.** Maternal Demographics and Infant Outcomes Between Partners Who Were Treated and Those Who Were Untreated

Characteristic	Total	Partner Not Treated (n = 55)	Partner Treated (n = 76)	P Value
Age, median (min, max)	25 (15–50)	25 (15–50)	25.5 (17–41)	*P* = 0.15625
Relationship status				
Married/cohabiting	117 (89.3%)	43 (78.2%)	74 (97.4%)	*P* = 0.0005
Single/separated/Divorced	14 (10.7%)	12 (21.8%)	2 (2.6%)	*P* = 0.0005
Participant HIV status				
Negative	85 (64.9%)	35 (63.6%)	50 (65.8%)	*P* = 0.955
Positive	41 (31.3%)	18 (32.7%)	23 (30.3%)	*P* = 0.955
Highest level of education				
No formal schooling	3 (2.3%)	1 (1.8%)	2 (2.6%)	*P* = 0.549
Any primary education	81 (61.8%)	37 (67.3%)	44 (57.9%)	*P* = 0.549
Any postprimary education	47 (35.9%)	17 (30.9%)	30 (39.5%)	*P* = 0.549
Number of ANC visits				
<3	35 (26.7%)	18 (32.7%)	17 (22.4%)	*P* = 0.1877
≥3	96 (73.3%)	37 (67.3%)	59 (77.6%)	
Prior miscarriage				
Yes	22 (16.8%)	6 (10.9%)	16 (21.1%)	*P* = 0.1268
No	109 (83.2%)	49 (89.1%)	60 (78.9%)	
Adverse birth outcome	13 (9.9%)	4 (7.3%)	9 (11.8%)	*P* = 0.3898
Stillbirth	2	1	1	
LBW	5	3	2	
Prematurity	6	0	6	

## QUALITATIVE RESULTS

Forty participants took part in semistructured interviews, including 21 HCWs (nurses and midwives, clinical officers, community midwives’ assistants, HIV diagnostic assistants, and medical assistants) and 19 women who were seropositive for syphilis.

### Flexible Process of PN

There were 4 approaches to PN identified: (1) participant-led PN (delivered by the women), (2) provider-assisted PN, (3) written PN, including the use of slips or health passports, and (4) male partner attendance during initial ANC to test both partners together. Providers identified participant-led PN as the default method:

*“…. we have never thought of exploring other options of notifying their husband we just tell them [the women] the results of the test and that they should notify their husband, if they can’t, they should come with him here for notification. So it’s just verbal, we have never given them any notification slip.” -Health Care Worker_Site 1.*

To facilitate PN, HCWs sometimes advised women to ask their partners to attend the clinic without disclosing the specific reason, allowing the HCW to lead the conversation. This approach was particularly common when women expressed fear of their partner’s reaction. In more complex situations, such as when a woman had multiple partners or a partner outside of marriage, HCWs took a more active role in the notification process. As one participant noted:

“...when the woman has several partners, we have an index where they leave phone numbers and we call them for the issue and for those married we ask them to tell their husbands and they come for us to explain the issue to them because some women end up being beaten by their husband,” -Health Care Worker_ Site 2.

Several HCWs described using written records or slips detailing the results and providing information on testing to be shared with their partner. Alternatively, some HCWs described noting the diagnosis in the woman’s health passport and asking her to explain the importance of treating their partner. Some noted that health passports were preferable to separate written notification because they were less likely to get lost. However, there were concerns around the confidentiality and privacy of this approach from some women:

“Because there are some things that are supposed to be confidential, so we can just assume that I was not happy because a health passport is touched by many people”. – Pregnant woman_Site 2.

### Barriers to PN and Treatment

Three main barriers to PN and treatment emerged.

#### Fear and Hesitancy of PN

Some participants reported fear of disclosing a syphilis diagnosis to their partners, particularly where this could reveal external sexual relationships, while others, despite uncertainty regarding infection source, remained concerned about potential negative partner reactions, limiting PN.

“I thought that he would shout at me and not understand it, but he was full of understanding, then we came together here…I thought that he would assume a lot about me, which is not the case” – Pregnant woman_ Site 3.

Some women anticipated partner refusal of the diagnosis in the absence of symptoms, with perceived partner skepticism reducing the likelihood of notification. One participant from site 2, who completed PN but not testing noted, “He says that his body is good, it is able to cleanse anything.” When women believed their partner would not report to a clinic for testing and treatment, they reported they were less likely to notify their partner.

#### Men’s Unwillingness to Access Care

Most women indicated that despite PN, their husbands were unwilling or avoided seeking care. For instance, a participant from Site 1 reported:“…my husband always answers me that he cannot come to the hospital because he is busy, so I just ignore it, that [his treatment] is something without progress.” Similarly, a participant from Site 2 reported: “I already explained to him that the nurse says we should go to the hospital together, but he refuses.”

Some partners cited financial constraints as barriers to care, while others, particularly those working abroad, were unwilling to seek testing or treatment in their host setting.

#### Communication and Misinformation

A key barrier in the PN and treatment process identified by HCWs and women was communication and knowledge sharing with partners, which could lead to misinformation. As one HCW explained,

“It is better that women come with their spouses because when they come together, there are fewer queries as compared to when they visit alone.” - Health Care Worker_Site 3. Another emphasized,

“…many men are never willing [to get tested]. Maybe sometimes the women don’t explain properly that’s why sometimes we prefer that they should just tell them to come together that we should explain to them.” - Health Care Worker Site 3.

Gendered power dynamics also affected the partner-led notification process. Traditionally, some men resisted taking directions from their wives, thus disregarding their notifications regarding testing and treatment.

“You know men look down on women, when telling them, they feel like women can’t tell them anything even though most times they don’t come.” – Health Care Worker Site 3.

This poor communication or lack of information can influence the decision-making or actions of partners to seek testing or treatment, as explained by another HCW:

“If they were coming together at antenatal during the first visit we would have been doing the counselling together but hearing from their wives may be a little disturbing depending on how she delivered the information and accepting the treatment becomes hard because they got the wrong information in the first place.” – Health Care Worker_Site 1.

## DISCUSSION

This study found that attrition from the PN cascade was commonplace and occurred both at the point of partner testing and partner treatment, several themes from the qualitative data helped to explain these findings. Firstly, there was evidence that attrition was partially due to inconsistent messaging from providers about what was required. Providers themselves identified poor education and stigma specific to syphilis in pregnancy as a barrier for care delivery. Secondly, we found attrition was higher at the point of treatment, which has also been reported in a South African study of syphilis in pregnancy, which reported notification at 93% but treatment at only 63%.^26^ By contrast, rates of PN and partner treatment reported in a systematic review of studies of all STI, across all treatment settings from sub-Saharan Africa, were much lower at only 53% for PN and 25% partner treatment.^27^ This observation may indicate higher intrinsic motivation in the setting of pregnancy as was mentioned by participants in this study. Pregnant women in this study frequently reported fear of notifying their partners, which adversely affected partner testing, a finding which is common in other settings.^27,28^ Where women did inform their partners, the costs associated with care seeking were a recurrent issue affecting partner treatment. This finding is consistent with the literature from other Southern African countries but it is notable that financial incentives alone have been unsuccessful in improving uptake.^17^ Utilizing key aspects of couples’ voluntary counseling and testing methods from HIV programmes may support the development of couple-based counseling for syphilis in pregnancy, with our qualitative data further supporting the acceptability and feasibility of this approach. Our findings support the need for multi-level interventions which improve education, reduce stigma, address access constraints, and empower flexible provider approaches to attain EMTCT targets in Malawi.

There was no association between maternal age or education level and successful PN in this study, which contrasts with other studies in similar settings.^26^ Of note, being married or cohabiting with a partner was clearly protective factor for successful PN in this study and our qualitative data provide several potential reasons for this. Firstly, HCWs clearly identified that direct provider-led communication, which was not the standard, was particularly important in complex relationship situations or where women felt unsafe. Fostering this approach may be essential in reaching partners of the most vulnerable women. A tailored partner-notification pathway for women in high-risk groups, such as adolescents and female sex workers, may enhance intervention effectiveness and improve health outcomes. Secondly, women reported that despite notification and access, partners were often reticent to attend ANC with them. The unwillingness of partners to attend antenatal clinics for treatment has also been reported previously in both Uganda and Kenya.^28,29^ Standardized male partner involvement in ANC is recommended by the Malawi Ministry of Health, participation remains variable (19%–64%).^24^ While provider referral or expedited partner treatment at ANC may overcome access barriers for couples willing to engage in PN, it is likely that broader community sensitization strategies to increase male involvement in ANC or strategies that provide for partners unwilling to attend ANC may be crucial in some instances. This study suggests that systems which can provide for flexible, provider-led approaches and are tailored to an individual’s needs may be both more acceptable and more effective in improving PN coverage.

To our knowledge, this is the first study in Malawi to report on PN specific to syphilis in the antenatal setting. The study’s mixed methods design enabled triangulation of quantitative coverage data with in-depth qualitative insights from both HCWs and pregnant women, allowing identification of both facilitators and barriers to PN in this setting. In this study, we propose a definition for PN, partner treatment (any), and appropriate PM. While partner treatment without prior testing may be justified in settings with high positivity rates, this approach risks overtreatment and associated costs, underscoring the need for context-specific evaluations to determine optimal strategies across different populations and risk groups. Ultimately, successful PM in the context of syphilis in pregnancy is a strategy that prevents maternal reinfection in pregnancy while meeting the clinical needs of partners. This concept should be addressed in the design of future studies of PN in this context, including the choice of outcome evaluation. Limitations included the lack of perspective of male partners within this study. The quantitative component was limited to a periurban and urban setting in a single region of Malawi and was facility-based and time-bound, which may restrict the generalizability. In addition, data relied on participant self-report, which may be subject to recall and social desirability bias.

PN is a critical component of antenatal syphilis management, with the World Health Organization recommending partner screening and treatment to reduce mother-to-child transmission.^30^ Yet evidence, particularly from low- and middle-income settings such as Malawi, remains limited and heterogeneous due to inconsistent definitions of coverage versus effectiveness. There is an urgent need for standardized, outcome-oriented operational definitions of PN in pregnancy and for well-designed prospective implementation studies to identify strategies that effectively overcome barriers at the index case, partner, and health-system levels.

Acknowledgments: The authors would also like to acknowledge the staff and patients working in and attending the health centers for their support during the course of this study.

## Notes

1
*The DIPLOMATIC Global Health Group Collaborating author include Monica Patricia Malata, Catherine Bamuya, Chifundo Kondoni, Gladys Membe Gadama, and David Lissauer.*
